# A Refined Hot Melt Printing Technique with Real-Time CT Imaging Capability

**DOI:** 10.3390/mi13101794

**Published:** 2022-10-21

**Authors:** Kirsty Muldoon, Zeeshan Ahmad, Yu-Chuan Su, Fan-Gang Tseng, Xing Chen, James A. D. McLaughlin, Ming-Wei Chang

**Affiliations:** 1 Nanotechnology and Integrated Bioengineering Centre (NIBEC), University of Ulster, Belfast BT15 1ED, UK; 2 School of Pharmacy, De Montfort University, Leicester LE1 9BH, UK; 3 Department of Engineering and System Science, National Tsing Hua University, Hsinchu 300044, Taiwan; 4 Institute of NanoEngineering and MicroSystem, National Tsing Hua University, Hsinchu 300044, Taiwan; 5 Department of Engineering and System Science, Frontier Research Center on Fundamental and Applied Sciences of Matters, National Tsing Hua University, Hsinchu 300044, Taiwan; 6 Key Laboratory for Biomedical Engineering of Education Ministry of China, Zhejiang University, Hangzhou 310027, China

**Keywords:** 3D printing, CT imaging, encapsulation, control release, micropore

## Abstract

Personalised drug delivery systems with the ability to offer real-time imaging and control release are an advancement in diagnostic and therapeutic applications. This allows for a tailored drug dosage specific to the patient with a release profile that offers the optimum therapeutic effect. Coupling this application with medical imaging capabilities, real-time contrast can be viewed to display the interaction with the host. Current approaches towards such novelty produce a drug burst release profile and contrasting agents associated with side effects as a result of poor encapsulation of these components. In this study, a 3D-printed drug delivery matrix with real-time imaging is engineered. Polycaprolactone (PCL) forms the bulk structure and encapsulates tetracycline hydrochloride (TH), an antibiotic drug and Iron Oxide Nanoparticles (IONP, Fe_3_O_4_), a superparamagnetic contrasting agent. Hot melt extrusion (HME) coupled with fused deposition modelling (FDM) is utilised to promote the encapsulation of TH and IONP. The effect of additives on the formation of micropores (10–20 µm) on the 3D-printed surface was investigated. The high-resolution process demonstrated successful encapsulation of both bioactive and nano components to present promising applications in drug delivery systems, medical imaging and targeted therapy.

## 1. Introduction

Hot melt extrusion (HME) is a form of 3D printing that applies high pressure through thermoplastic polymers at a critical temperature [1]. The process is often coupled with fused deposition modelling (FDM) to manufacture 3D designs by depositing molten material layer by layer to form a geometry sourced from design software [2]. HME and FDM have been employed in many applications such as pharmaceutical [3], biomedicine [4] and sensors [5]. Three-dimensional printing has progressed over the years with more recent methods including inkjet, stereolithography and electrohydrodynamic printing which are capable of producing structures at a highly precise micro and nano scale [6,7,8]. Due to these emerging technologies, HME/FDM is often overlooked as a technique that offers resolution appropriate for the aforementioned applications. Cui et al. reported that FDM can in fact offer a better resolution than other technologies, with greater advantages in finer structures and shapes [9]. This claim is supported through this study as we discuss a microscale resolution in geometry and surface morphology portraying micropores.

HME coupled with FDM is a favourable choice of additive manufacturing as it is a solvent free process, which therefore, does not require additional drying steps, enabling continuous production as a result [10]. A challenge to the manufacturing process is the diversity of materials as it is mostly limited to polymers. The polymers must be carefully selected according to their amorphous or semi-crystalline nature and their crystallization temperatures [11]. Ideally, amorphous polymers are most suitable as they solidify quickly with minimal shrinkage which is essential for layer-by-layer printing to allow the uppermost layer to ‘stick’ to the previous [11]. Most polymers are thixotropic; therefore, an optimal processing temperature is required to ensure a low viscosity of the printing ink [12]. Pressure is another critical parameter that is necessary for the extrusion of molten material. Its optimization may require alterations according to the viscosity of the polymer used, where a highly viscous polymer requires higher pressure to extrude it from the printing nozzle [13]. A manufactured high-quality product from HME/FDM requires the optimization of these printing parameters for successful extrusion and surface finish.

There is an enormous interest in pharmaceutical fabrication via 3D printing due to the ability to develop personalized medicine with tailored therapeutic properties [14,15,16,17]. The advantage of a personalized drug delivery system (DDS) over conventional methods of tablet consumption is that it does not operate on a one size fits all approach. Often, mass manufactured tablets have an administered dose that is beyond the optimum and does not consider individual patient requirements, therefore resulting in toxic effects [18]. Personalized medicine produced via 3D printing improves product complexity and on-demand manufacturing that can be tailored according to body mass index, metabolism, genetic disorders and a range of release profiles [10]. Other 3D techniques are capable of producing personalized drug delivery systems, but due to solvent-based processes, this produces drug release profiles that exhibit a burst release [19]. This is not always advantageous as this type of delivery is controlled via first pass metabolism, which does not always remain in the therapeutic window. This issue is avoided in this research as the physical mixture of drug and polymer produces a monolithic drug delivery system via HME/FDM by encapsulating the drug within the polymer to prevent a burst release profile and produce a sustained profile as a more admirable alternative [20]. The design of DDS via HME/FDM is difficult due to most drugs being thermolabile, therefore hindering the application possibilities of the 3D printing process [10]. Thermosensitive drugs require incorporation into a blend in order to prevent the decomposition of active pharmaceutical ingredients (API), which can be achieved through the monolithic system [12]. DDS has been developed in many forms such as microfluidic channels, in addition to the 3D-printed model in this study, as an alternative form of drug delivery and offers rapid and cost-effective approaches [21]. Both microfluidic and 3D-printed models can operate via a flow focusing mechanism that involves a controlled liquid interface for nanoprecipitation of particles [22]. The 3D-printed DDS structure is comparable to other methods of DDS. Amoyav et al. demonstrated a microfluidic device to achieve a sustained release profile, which is similar to the findings based on the 3D-printed structure [23]. 

Implantable DDS is a beneficial delivery alternative due to direct delivery to the target tissue and the avoidance of toxic side effects associated with systemic administration [20]. However, once the device has been inserted, the effect on the host environment is unknown. Therefore, a suitable contrasting agent (CA) is required to view the location of the structure and histology of the surrounding environment. Conventional contrasting agents typically include iodine, which is administered intravenously and can cause toxicity due to exposure to the circulatory system and cause rapid kidney excretion [24]. In the case of CT (computed tomography) imaging, conventional CAs have low efficiencies due to their energy co-efficient being outside the voltage range used in medical imaging [25]. Currently, CT imaging, alongside MRI (magnetic resonance imaging) and ultrasound are popular and clinically available imaging techniques that allow for non-invasive diagnosis by employing data based on diffusion, inflammation, and angiogenesis [26,27,28]. In addition, CT imaging of DDS provides detailed 3D images of morphology, which displays the relationships between delivery device formulation, performance and structure [28]. Iron oxide nanoparticles (IONP) have low cytotoxicity, colloidal stability and superparamagnetic properties that enhance contrasting ability but show limited understanding of printed matrix for CT imaging [25]. As an improved alternative, iron oxide nanoparticles are encapsulated within PCL alongside TH to produce a local diagnostic real-time imaging device with fundamental requisites for biomedical applications. 

In this study, a novel drug delivery system with real-time imaging capabilities is engineered at a high resolution (225–425 µm) with microporous features (10–20 µm) via HME coupled with FDM. The bulk printing ink comprises FDA-approved PCL due to its biocompatibility and its thermoplastic and amorphous properties, making it a suitable encapsulating material. Tetracycline hydrochloride (TH) is selected as a result of its broad therapeutic function and high melting point of 220–223 °C [29], which is beyond the optimal temperature parameter of PCL. The thermal stable nature of TH makes it an appropriate drug of choice as it remains stable in this study. Iron oxide nanoparticles were uniformly incorporated into the printing ink to highlight the contrast between density variations associated with the 3D-printed structure and its surrounding environment. The results investigated in this study include the optimization of PCL printing parameters, printed structure, surface morphology, chemical composition, surface wettability and tensile properties alongside CT imaging and drug release profile of the encapsulated TH. 

## 2. Materials and Methods

### 2.1. Materials

Polycaprolactone powder (PCL, Mw = 50,000 gmol^−1^) was purchased from Polyscience Europe (Germany). Tetracycline Hydrochloride (TH, Mw = 480.90 gmol^−1^) was purchased from Sigma Aldrich, USA, and iron oxide nanoparticles (IONP) (Fe_3_O_4_, particle size < 63 nm) were obtained from Inoxia chemicals (UK). 

### 2.2. 3D Printing Fabrication 

PCL printing parameters optimization and multifunctional samples containing TH and IONP were produced using a stainless-steel syringe equipped with a 22 gauge (internal diameter 0.4 mm) needle tip loaded in a high-temperature printing cartridge of 3D-Bioplotter (EnvisionTEC, Gladbeck, Germany). Thirty min was allowed for sufficient melting of the printing ink prior to extrusion for all samples printed at 130 °C, and the optimization temperature was investigated between 100 and 130 °C. The design for all samples in this study is a 1 cm by 1 cm square grid with a 0°/90° pattern to produce square shaped pores. The printing parameters and geometry selection were operated using VisualMachine 2.8.115 software (EnvisionTEC, Gladbeck, Germany). Samples prepared according to Table 1 were dry mixed prior to loading into the stainless steel syringe. The concentration of TH and IONP was in relation to the success of existing studies.

### 2.3. Surface Morphology

Structure and surface features were analyzed by Scanning Electron Microscopy (SEM) on all 3D-printed samples produced in this study. Hitachi SU5000 field emission instrument (Hitachi, Europe) was used in high vacuum with secondary electron (SE) signal. All images were captured with an accelerating voltage of 2 kV and spot intensity at 10. Images were captured at a low magnification of ×50 to allow for a greater area of the 3D printed structure to be analyzed for uniformity. High magnification images were captured to observe surface topography and to identify micropores. The fiber diameter and void distance were calculated using ImageJ software. Fifty measurements were taken for both parameters to form an average size distribution. Micro CT images were used to conduct a color analysis of the 3D-printed sample using ImageJ.

### 2.4. Chemical Composition

Fourier transform infrared (FTIR) spectroscopy was used to analyze the individual powder components of the sample prior to printing. Attenuated total reflectance (ATR-FTIR) was conducted on 3D-printed samples as its structure could not be ground to powder to incorporate with KBr background. As the detection of the components were required to compare chemical composition, typical 3D-printed samples were examined in transmission mode using Varian 640-IR (UK) at a resolution of 4 cm^−1^ recorded within a wave range of 400–4000 cm^−1^ with 30 scans per sample.

To identify the encapsulation ability of the 3D printing process, energy dispersive X-ray (EDX) was conducted to identify the elements present on the surface of the printed sample. Hitachi SU5000 field emission instrument (Hitachi, Europe) was employed with an accelerating voltage of 2 kV to form an elemental mapping of the surface. 

### 2.5. Surface Water Contact Angle

The surface wettability of 3D-printed PCL and samples containing Fe_3_O_4_ and TH was studied using water contact angle (WCA) measurements using an optical contact angle meter CAM 200 (KSV instruments LTD, Helsinki, Finland). Measurements were performed with a 10 µL drop out size, 20 µL/s drop and dispense rate. Three repeats were taken per sample; each measurement consisted of a left and right WCA. Microsoft Excel was used to calculate the average of left and right WCA. Subsequently, the value was then used per repeat to obtain an overall average WCA for each sample. 

### 2.6. Tensile Testing

Three-dimensionally printed samples (20 mm width × 0.36 mm thickness) were tested using Instron 5500R Mechanical Testing apparatus (Instron, Norwood, MA, USA). A load cell of 2 kN was used, and crosshead speed was set at 10 mm/min until failure. Series IX version 8.33.00 was used to collect and process displacement and load results. This was converted to tensile stress (MPa) versus tensile strain (%) using Microsoft Excel. 

### 2.7. MicroCT

To investigate the contrasting abilities of IONP, X-ray microcomputed tomography (MicroCT) was performed on 3D-printed PCL with and without IONPs. The analysis was performed on samples by SkyScan 1275 (Bruker, Billerica, MA, USA) with 360° rotation, voltage source of 38 kV and current 25 µA. The CT images were reconstructed by slicing the 3D model using Bruker’s NRECON 2.0, and manual threshold of images was conducted using Brukers CTvox software.

### 2.8. Drug Release Study

To construct a standard curve, 10 solutions of TH in deionized water were created in the concentration range of 0.1–1.0 g/mL. The standard curve was produced by measuring the absorbance of TH at 360 nm according to previous work [30] using Lambda 365 UV-Vis Spectrophotometer (Perkin Elmer, Buckinghamshire, UK). The concentration of drug released from each sample was calculated by immersing the sample in 10 mL of deionized water with magnetic stirring at 200 rpm. Every 24 h, for 7 days, 10 µL was taken from the solution of the immersed sample and diluted in 100 µL to comply with the 1 in 100 dilutions of the standard curve. UV–Vis was conducted on this sample dilution and the absorbance was recorded at 360 nm. The absorbance values were substituted into the equation of the trendline of the standard curve to obtain a corresponding concentration of TH released from the 3D-printed structure.

## 3. Results and Discussion

### 3.1. The Effect of Controlling Parameters 

**Figure 1:**
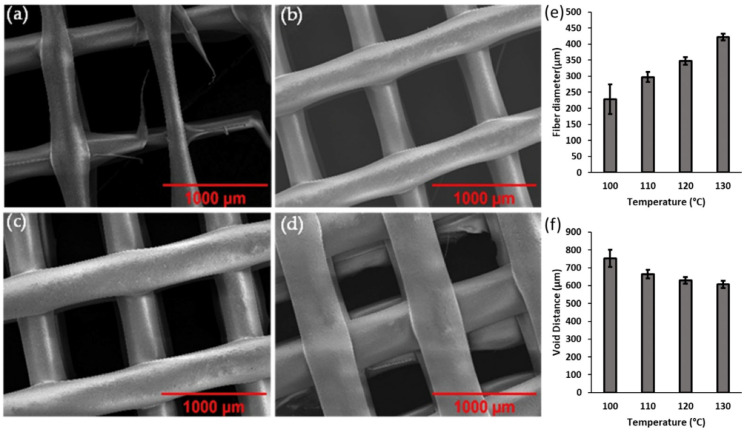
SEM images of 3D-printed PCL at (**a**) 100 °C, (**b**) 110 °C, (**c**) 120 °C and (**d**) 130 °C. Distribution of average size for (**e**) fiber diameter and (**f**) void distance at various temperatures.

The printing parameters of hot melt extrusion including temperature of the printing ink and system pressure play an important role in the microstructure formation. It is essential that these printing conditions are optimized as it also affects the overall quality and repeatability of the method that shows accuracy in the uniform of single fiber deposition and precision control in the final geometry. Hence, the effect of temperature and pressure on the printing of PCL was first investigated and optimized. The optimal parameters are determined by the standard deviation of measurements, final geometry and precision control, which indicates the uniformity of printed material and void spaces. The melting point of PCL is around 60 °C [31]; therefore, to ensure the PCL powder is in a suitable condition for printing, the parameter needs to be set around double the melting point of the material due to the conduction of heat required throughout the printing syringe to the entire PCL content contained inside. During the temperature analysis, the pressure was maintained at an optimal pressure of 6 bar as a controlled variable that does not alter the resultant printed sample. SEM images of PCL samples printed from 100 °C to 130 °C are presented in Figure 1a–d. Figure 1a displays the non-uniform printing of PCL material when the printing ink was set at 100 °C. This is due to the insufficient temperature of the printing ink during printing, as it had not reached its minimum melting point uniformly. This printing parameter has a broad distribution in the fiber diameter of the printed PCL (Figure 1e), resulting in the largest standard deviation at a value of ± 46, suggesting that the extrusion of PCL at 100 °C is unstable. 

The void distance, defined as distance between the printed fibers, shows critical parameters for different applications. For example, the effect on mechanical properties [32], manipulation of drug release rate [33] and control of cell migration [34]. The void distance is large as represented in Figure 1f, as a reflection of the fiber diameter measuring an average of 227.8 µm caused by insufficient melting and extrusion of PCL. Increasing the temperature to 110 °C and 120 °C microstructure presents an improvement in the uniformity of extrusion as shown in the SEM image in Figure 1b,c. The fiber diameter of extruded PCL increases with increasing temperature, while the void space diameter decreases simultaneously (Figure 1e,f). In addition, the standard deviation has decreased to ±16 and ±11 respective to 110 °C and 120 °C, therefore suggesting that these temperatures are much more suitable for achieving uniform microstructure printing. The uniformity of fiber diameter and void distance seen in Figure 1d is supported by a small standard deviation represented in Figure 1e,f, conveying that 130 °C is the optimal temperature for printing of PCL microstructures. 

**Figure 2:**
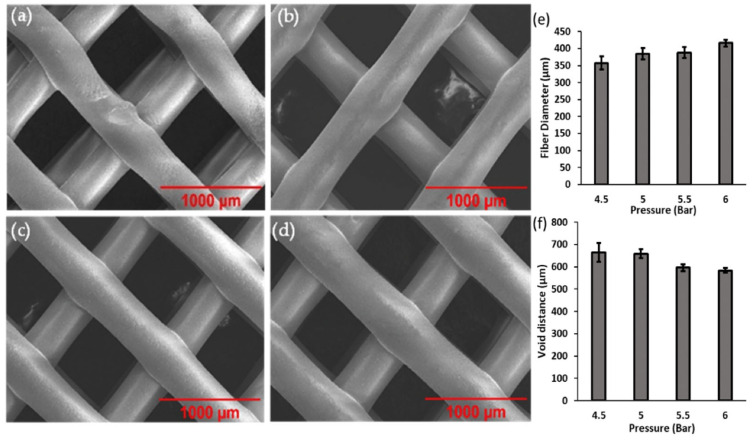
SEM images of 3D-printed PCL at (**a**) 4.5 bar, (**b**) 5.0 bar, (**c**) 5.5 bar and (**d**) 6.0 bar. Distribution of average measurement for (**e**) fiber diameter and (**f**) void distance at various pressures.

The pressure at which the printing ink is extruded through the printing nozzle can largely affect the resultant product. If the extrusion pressure is too low, there is not enough force to drive the molten ink through the orifice of the printer cartridge. Alternatively, if the pressure is too high, this can cause excess ink extruding and therefore altering the precision and dimensions of the print. Therefore, the pressure required for optimal results was studied in the range from 4.5 to 6 bar while keeping the temperature parameter constant at its optimal setting of 130 °C. The SEM images in Figure 2a–d show a general overall maintained structure. However, Figure 2a displays an irregular surface and straightness of printed PCL at the lowest pressure of 4.5 bar. In comparison to Figure 2c,d, the surface smoothness improves and the geometry appears more controlled when extrusion pressures were performed from 5 to 5.5 bar. It is obvious from Figure 2d that the diameter of the printed ink is greater than those printed below 6 bar. The data presented in Figure 2e,f demonstrate that the sample printed at 6 bar has the largest fiber diameter with an average value of 416 µm and smallest void distance average of 583 µm, while also possessing the smallest standard deviation result of around ±10.5 µm. Hence, this parameter proves to be the most reliable and highly precise, therefore concluding that 6 bar is the optimal pressure parameter for printing PCL microstructures.

### 3.2. The Effect of Bioactive Encapsulation

To further study the encapsulation of bioactive in the optimized printing parameters, TH and IONPs were added to the printing ink comprising of bulk material PCL. Incorporating these significantly different materials into the printing ink for HME demonstrates the material diversity and application opportunities. The concentrations of TH were determined by Wang et al. and Turan and Metin et al. who incorporated TH in previous studies in the ranges of 1–2 w/w% and 0.5–5 w/v%, respectively, as a suitable dosage form for therapeutic applications [35,36] The concentration of IONPs was kept at a constant low concentration of 0.5 wt%, which is within the range of existing studies. Ko et al. included 5 wt% of IONPs in their study to form a magnetic field sensitive hydrogel [37]. However, three samples with varied TH drug concentrations were designed in the current work, and their contents are displayed in Table 1. 

**Figure 3:**
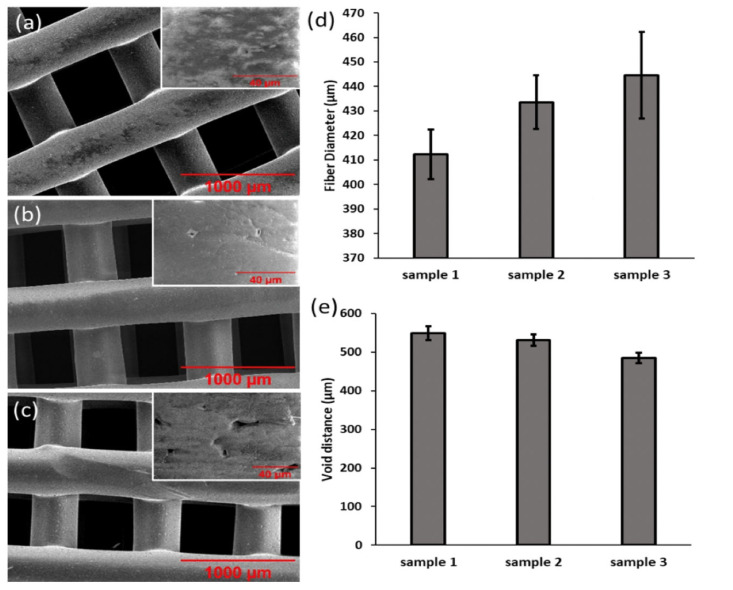
SEM images of 3D-printed (**a**) sample 1, (**b**) sample 2 and (**c**) sample 3 with insets of surface morphology. Distribution of average size of (**d**) fiber diameter and (**e**) void distance.

SEM images of the three samples in Figure 3a–c present that varying the concentration of TH does not affect geometry of the printed sample. However, there is a difference between each sample in both void distance and fiber diameter, as shown in Figure 3e,f. The trend in results implies that, as the concentration of TH increases, the fiber diameter increases from 412 µm to 434 µm then to 445 µm with sample 1, 2 and 3, respectively. Meanwhile, void diameter decreases from 549 µm in sample 1 to 531 µm in sample 2 and then to 485 µm in sample 3. These findings coincide with a study by Karuppuswamy et al., who recorded similar results obtained by the electrospinning method, that fiber diameter increased with increasing TH concentration [38]. Additionally, another noticeable trend occurring with increasing TH concentration is the larger quantity of micropores on the sample surface, which is shown in the inset images of Figure 3a–c. The micropores measure approximately 10 µm in sample 1; this increases to approximately 20 µm by sample 3 due to the increase in TH concentration. This phenomenon also occurred in previous studies that seen the introduction of TH into the printing ink. Previous work also showed morphological changes under SEM with the loading of TH with an enhanced surface roughness in electrospun nanofibers [38]. Mathew et al. utilized fused deposition modelling of TH, which presented a degree of roughness and surface defects with increasing TH concentrations [39]. The micropores may be a result of chemical interactions that occur during HME, given that TH has a first-order reaction kinetic at 130 °C, and the chemical interactions that occur, and therefore, formation of micropores is dependent on concentration [29].

### 3.3. FTIR and EDX

**Figure 4:**
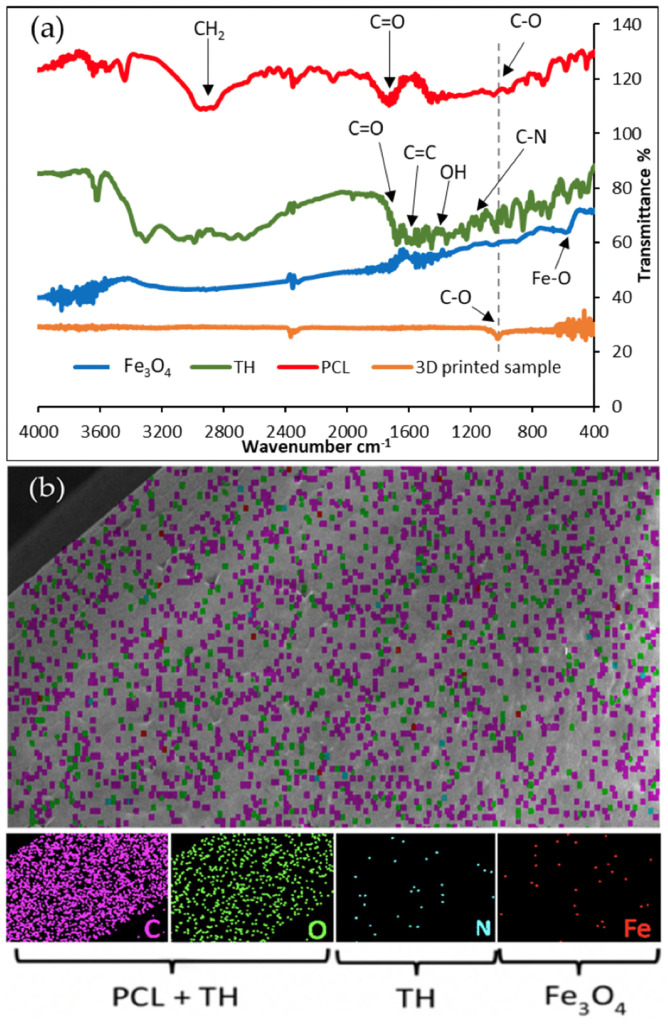
Characterization of samples. (**a**) FTIR of each component of the samples and 3D-printed sample and (**b**) EDX of 3D-printed sample.

To determine the successful encapsulation of TH and IONPs in the 3D-printed structure, FTIR and EDX were conducted and are presented in Figure 4. Figure 4a displays the transmittance spectra for each raw component in powder form that forms the 3D-printed sample and attenuated total reflectance (ATR) of the 3D-printed sample. Due to the structure of the 3D-printed sample, it could not be ground with a pestle and mortar into powder form for FTIR. Therefore, ATR was conducted as a result to form transmittance spectra. The characteristic peaks of pure PCL are observed at 2951 and 2862 cm^−1^, which represent methylene (CH_2_) groups and carbonyl groups (C=O) observed at 1714 cm^−1^ [8]. The TH spectra has peaks at 1614 and 1666 cm^−1^, which correspond to the stretching of aromatic ring C=C and C=O, respectively. OH bending is represented by a peak at 1454 cm^−1^ and 1228 cm^−1^ peak equates to C-N stretching and N-H bending in the transmittance of TH [35]. The FTIR spectrum of Fe_3_O_4_ has a typical characteristic peak which is at 573 cm^−1^, this is due to the presence of Fe-O bond [40]. The result for the 3D-printed sample presents a mostly flat spectrum with the exception of a peak at 1030 cm^−1^, which is characteristic of PCL corresponding to a shifted characteristic peak of PCL, suggesting that relatively lower concentrations of TH and IONPs are encapsulated within PCL.

Figure 4b displays a map of the elements detected on the surface of the printed sample. The most abundant elements on the EDS image are carbon and oxygen, which correspond to the chemical formula of PCL ((C_6_H_10_O_2_)_n_) [41]. Nitrogen is an elemental component of TH with the chemical formula C_22_H_24_N_2_O_8_.HCl [42], and Fe is contributed by Fe_3_O_4_. The distribution of elements represented by colored dots indicates that the preparation of the printing ink was effective as all components were evenly distributed throughout the structure. Given that no additional elements were detected, the resultant sample was free from impurities. The minute quantity of N and Fe detected depict their presence, as supported by the FTIR spectra.

### 3.4. Water Contact Angle and Mechanical Test

**Figure 5:**
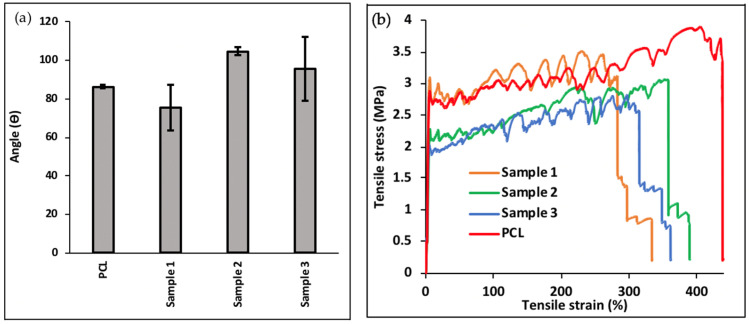
Analysis on 3D-printed samples. (**a**) Water contact angle results of 3D-printed samples and PCL, and (**b**) tensile test.

The 3D printed sample with customized water contact angle and mechanical property produced via HME is open to being employed in many different applications, e.g., tissue engineering sector and drug delivery systems. Figure 5a represents the results of water contact angle test conducted on 3D-printed samples. The addition of TH and IONPs produced a greater variation of results as the standard deviations of the three samples are much greater than that of pure PCL, therefore indicating that there is more variation to the hydrophilicity of the sample surface that contains TH and IONPs. Sample 1 had an average water contact angle that was smaller than that of PCL, which can be explained by TH’s hydrophilic nature [43]. As the concentration increases to 0.75 wt% in sample 2, the average WCA greatly increases to 105°. A similar trend was reported by Saha et al. who observed an increase in WCA with an increase in TH concentration [44]. The average angle does not continue to increase as the TH concentration is increased further to 1 wt% in sample 3. However, when the standard deviation and all measurements are taken into consideration, there is a positive correlation due to the TH concentration being lower than PCL used in the printed structure. 

The difference in mechanical properties of the 3 samples and pure PCL were investigated. Figure 5b shows a tensile stress–strain profile that appears to be in the form of a jagged line due to the individual fracture of vertically printed material. PCL exhibits the greatest tensile strength among all tested samples, with an ultimate tensile strength value of 3.9 MPa. Table 2 shows that the ultimate tensile strengths of samples 1, 2 and 3 are 3.5 MPa, 3.0 MPa and 2.8 MPa, respectively. This depicts a negative correlation trend: as TH concentration increases, the ultimate tensile strength decreases. This can be explained by the increase in micropores on the surface of the samples with increasing TH concentration, previously seen in Figure 3a–c. The presence of micropores may function as stress and strain concentrators, which result in the sample to fail and therefore to have a weaker ultimate tensile strength at a higher TH concentration. Similar to existing studies by Wang et al. and Lin et al., Figure 5b also presented PCL as having the greatest tensile strain in comparison to samples containing TH due to poor interfacial interactions and unstable phase dispersion in composite materials [35,45].

### 3.5. In-Vitro MicroCT Evaluation and Drug Release 

**Figure 6:**
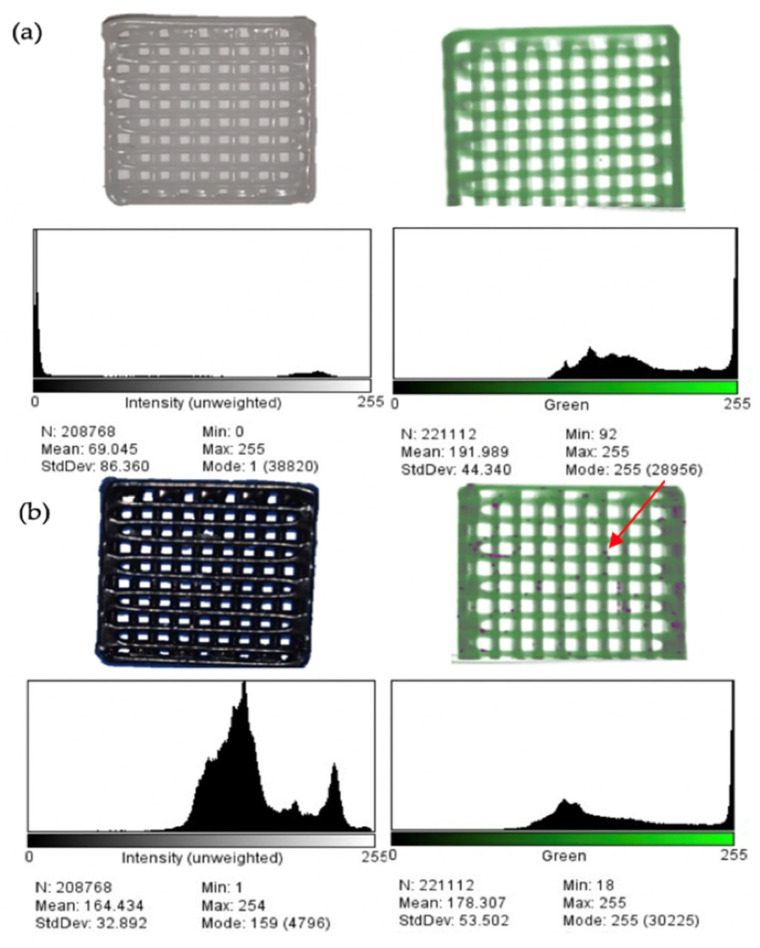

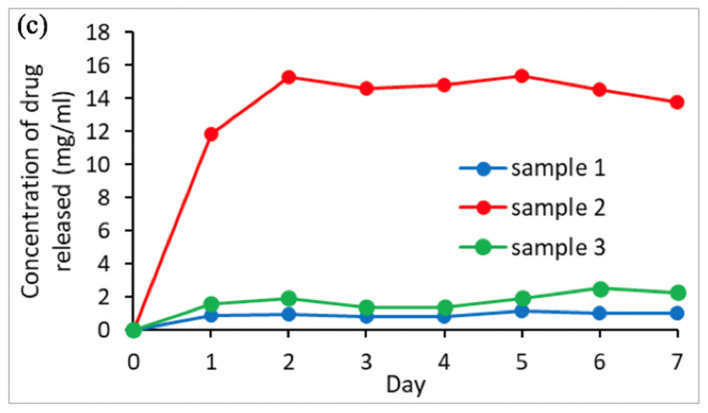
Images, MicroCT scan and histogram of particle distribution of 3D-printed (**a**) PCL and (**b**) PCL with Fe_3_O_4_. (**c**) Seven-day drug release profile.

The fusion of IONPs with PCL results in a contrasting difference compared to pure PCL. Figure 6a,b display a photograph of 3D-printed PCL without Fe_3_O_4_ and with Fe_3_O_4_ nanoparticles in the top left of each figure. The drastic difference in color is attributed to Fe_3_O_4_ nanoparticles, which show black in its natural form. To statistically signify the contrasting difference, a color measurement was conducted, which concluded that the printed sample of PCL without Fe_3_O_4_ nanoparticles was measured to be 99% white, whereas PCL with Fe_3_O_4_ nanoparticles is 37.34% white as a result of the IONPs outcasting white PCL and forms a predominantly black sample. The histograms below the photographs of the two samples correspond to the intensity of the contrast; for PCL, without Fe_3_O_4_ nanoparticles, there is minimal intensity and a mean value of 69 pixels, whereas there is a significant increase in contrast intensity for printed PCL with Fe_3_O_4_ nanoparticles, with a mean of 164 pixels. This difference implies a statistically supported contrasting difference as a result of the encapsulation of IONPs within PCL structures.

Given that the sample components in powder form prior to printing are mixed efficiently, the resultant Fe_3_O_4_ nanoparticles have uniform distribution across the printed sample, as seen by the naked eye in Figure 6b. As IONPs retain good magnetism, their distribution on the micro level can be analyzed with an applied x-ray source. The right side images in Figure 6a,b are microCT images of PCL without and with Fe_3_O_4_ nanoparticles, respectively. The former displays a green profile of PCL with a histogram below denoting the intensity of green detected. The latter sample portrays a predominantly green profile with some variations in color throughout. The representation of IONPs in the microCT image does not correspond to the photograph of sample with Fe_3_O_4_ nanoparticles. This is due to the detection limit of microCT, which can only identify nanoparticles that have agglomerated, and their size has transitioned to the micro scale. This is viewed in Figure 6b by the highlighted purple regions indicated by the red arrow. Additionally, the corresponding histograms of the microCT images indicate a contrasting difference between the two samples as the green profile intensity, representing PCL, decreases with the inclusion of IONPs. The contrasting IONPs from the microCT are dispersed throughout the sample, implying that the preparation and HME of the ink is successful in encapsulating IONPs within PCL polymer from powder form to final structure. These results indicate the contrasting capabilities of IONPs, which are suitable to applications including medical imaging [46], magnetic hyperthermia treatment [47] and drug carriers [48].

TH has been extensively used as an antibiotic in drug delivery systems due to its broad spectrum therapeutic capabilities [24,27]. Figure 6c shows TH release profile formed from printed structures using UV–VIS spectroscopy during a 7-day study. Each sample displays a sustained release profile, unlike typical burst release profiles, which are reported for drug delivery studies using TH [49]. Price et al. reported an approximate 90% release within 30 h of TH from a polymeric carrier. The encapsulation of TH within PCL via HME in this study allows for the drug release to be minimal and therefore enables the drug delivery system to operate for a longer period of time. Sample 2 exhibits the largest amount of drug release averaging at 14.32 mg/mL, whereas sample 1 and sample 3 exhibit average drug releases of 0.93 and 1.85 mg/mL, respectively. The high release from sample 2 is due to micropores on the sample surface and its geometry. Goyanes et al. reported a study on the effect of geometry on drug release from HME samples, suggesting that drug releases from the samples were dependent on the surface area to volume [50]. In comparison to reported research, the release profile of TH depends on the nature in which TH is contained within the DDS. Turan et al. reported a cumulative drug release of loaded TH copolymer/gelatin nanofibrous membranes. The results had shown that the trend between TH concentration and release percentage was a negative correlation: as the TH concentration increased, the release percentage decreased [36]. In contrast, Wang et al. reported a directly proportional relationship between increasing concentration and the release of TH from encapsulated fibers of PCL/PVP in the form of patches [35]. However, preparation of PCL containing Fe_3_O_4_ nanoparticles and TH drugs via HME coupled with FDM is promising for the development of 3D-printed matrices for CT imaging and drug delivery.

## 4. Conclusions

A high precision 3D-printed drug delivery system with real-time imaging capabilities was demonstrated via HME coupled with FDM. The temperature and pressure printing parameters of PCL was optimized prior to incorporating multifunctional components. The defined optimal parameters were concluded to be 130 °C and 6 bar, which were the set parameters for the continuation of the study. The addition of 0.5 wt% IONP had no adverse effect on the surface morphology of the structure however by increasing the concentration of TH from 0.5–1 wt% the quantity and size of micropores on the sample surface increased (from 10 to 20 µm). FTIR and EDX confirmed the encapsulation of TH and IONP, as PCL was the predominant detected material on the sample surface. Compared to PCL, the three 3D-printed samples showed a variation in surface wettability; however, when considering the outlying data, the trend implied that hydrophobicity of the sample surface increased with increasing TH concentration. PCL exhibited the greatest tensile strength compared to samples containing TH and IONP due to their addition resulting in micropores on the surface. The novel application presented a sustained TH drug release over 7 days as a result of the encapsulation of the drug, offering therapeutic benefits over a prolonged period of time. In addition, IONP presented micro level contrasting ability distributed homogeneously throughout the structure, which is advantageous as an alternative diagnostic tool. The combination of the printing process and diversity of materials demonstrates the fabrication of a multifunctional system to develop applications in personalized medicine.

## Figures and Tables

**Table 1:** Sample contents and their printing conditions.

	PCL (wt%)	Fe_3_O_4_ (wt%)	TH (wt%)	Temperature (°C)	Pressure (bar)
**Sample 1**	99	0.5	0.5	130	6
**Sample 2**	98.75	0.5	0.75	130	6
**Sample 3**	98.5	0.5	1.0	130	6

**Table 2:** Tensile strength and strain of 3D-printed samples.

3D Printed Sample	Tensile Strength (MPa)	Tensile Strain (%)
**PCL**	3.9	439
**Sample 1**	3.5	334
**Sample 2**	3.0	390
**Sample 3**	2.8	362
